# Adrenal Mitochondria and Steroidogenesis: From Individual Proteins to Functional Protein Assemblies

**DOI:** 10.3389/fendo.2016.00106

**Published:** 2016-07-29

**Authors:** Andrew Midzak, Vassilios Papadopoulos

**Affiliations:** ^1^Research Institute of the McGill University, Montreal, QC, Canada; ^2^Department of Biochemistry, McGill University, Montreal, QC, Canada; ^3^Department of Pharmacology and Therapeutics, McGill University, Montreal, QC, Canada

**Keywords:** cholesterol transport, translocator protein, steroidogenic acute regulatory protein, voltage-dependent anion channels, cytochrome P450 enzyme system, mitochondria, endoplasmic reticulum

## Abstract

The adrenal cortex is critical for physiological function as the central site of glucocorticoid and mineralocorticoid synthesis. It possesses a great degree of specialized compartmentalization at multiple hierarchical levels, ranging from the tissue down to the molecular levels. In this paper, we discuss this functionalization, beginning with the tissue zonation of the adrenal cortex and how this impacts steroidogenic output. We then discuss the cellular biology of steroidogenesis, placing special emphasis on the mitochondria. Mitochondria are classically known as the “powerhouses of the cell” for their central role in respiratory adenosine triphosphate synthesis, and attention is given to mitochondrial electron transport, in both the context of mitochondrial respiration and mitochondrial steroid metabolism. Building on work demonstrating functional assembly of large protein complexes in respiration, we further review research demonstrating a role for multimeric protein complexes in mitochondrial cholesterol transport, steroidogenesis, and mitochondria–endoplasmic reticulum contact. We aim to highlight with this review the shift in steroidogenic cell biology from a focus on the actions of individual proteins in isolation to the actions of protein assemblies working together to execute cellular functions.

## Introduction

The cortex of the adrenal is the principal site of synthesis of vertebrate glucocorticoid and mineralocorticoid steroid hormones (1). These hormones are two of the five classes of steroid hormones which are indispensable for mammalian development and physiology, the remaining including the estrogens, progestins, and androgens (2). The steroidogenic capacity of the adrenal gland is highly compartmentalized, performed by specialized cells, organelles, and proteins. The biosynthesis of steroids has been authoritatively reviewed (3), and this manuscript looks to focus attention on the compartmentalization of steroidogenesis of the adrenal mitochondria. To this end, several topics will be covered, including adrenal tissue zonation, mitochondrial organellar organization, and macromolecular protein complexes, all contributing to the regulation and optimization of adrenal endocrine signaling. Special attention will be devoted to our increasing understanding of multiprotein assemblies in mitochondrial function.

## Adrenal Gland Zonation

**Figure 1:**
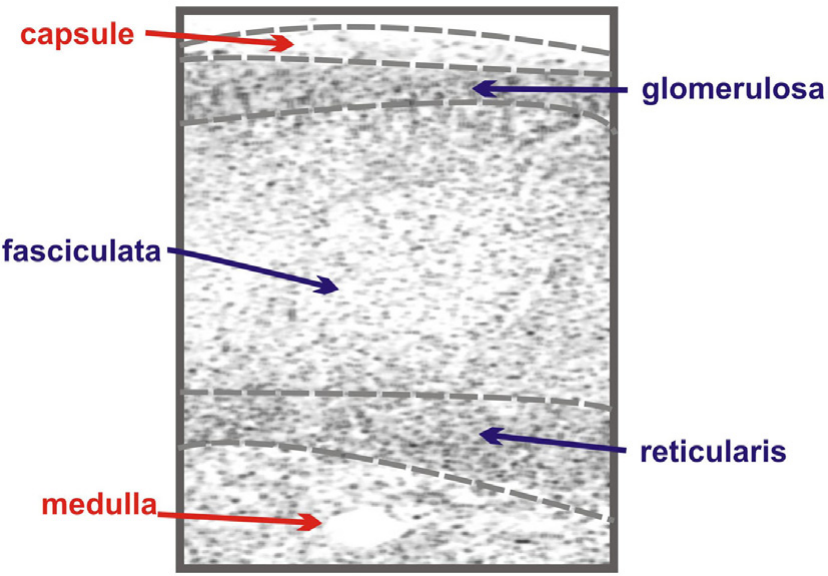
**Schematic of adrenal zonation**. The different functional zones of the human adrenal gland are depicted with the outermost capsule layer overlying the mineralocorticoid-synthesizing glomerulosa layer. The fasciculata layer lies under the granulosa layer and is responsible for the synthesis of the glucocorticoid cortisol. The final layer of the cortex, the reticularis, synthesizes the androgen dehydroepiandrosterone (DHEA), while the innermost layer of the schematic, the medulla, is composed of chromaffin cells, responsible for the production of the catecholamine epinephrine.

The adrenal gland is composed of two anatomically and functionally distinct compartments: the cortex and the medulla. The adrenal medulla is innervated with chromaffin cells and plays a key role in catecholamine synthesis and the sympathetic stress response (4), while the cortex contains the steroidogenic cells responsible for the adrenal’s contribution to the endocrine system (5). The adrenal cortex is further compartmentalized, in humans functionally and morphologically divided into three concentric layers: the zona glomerulosa, the zona fasciculata, and the zona reticularis (Figure 1).

**Figure 2:**
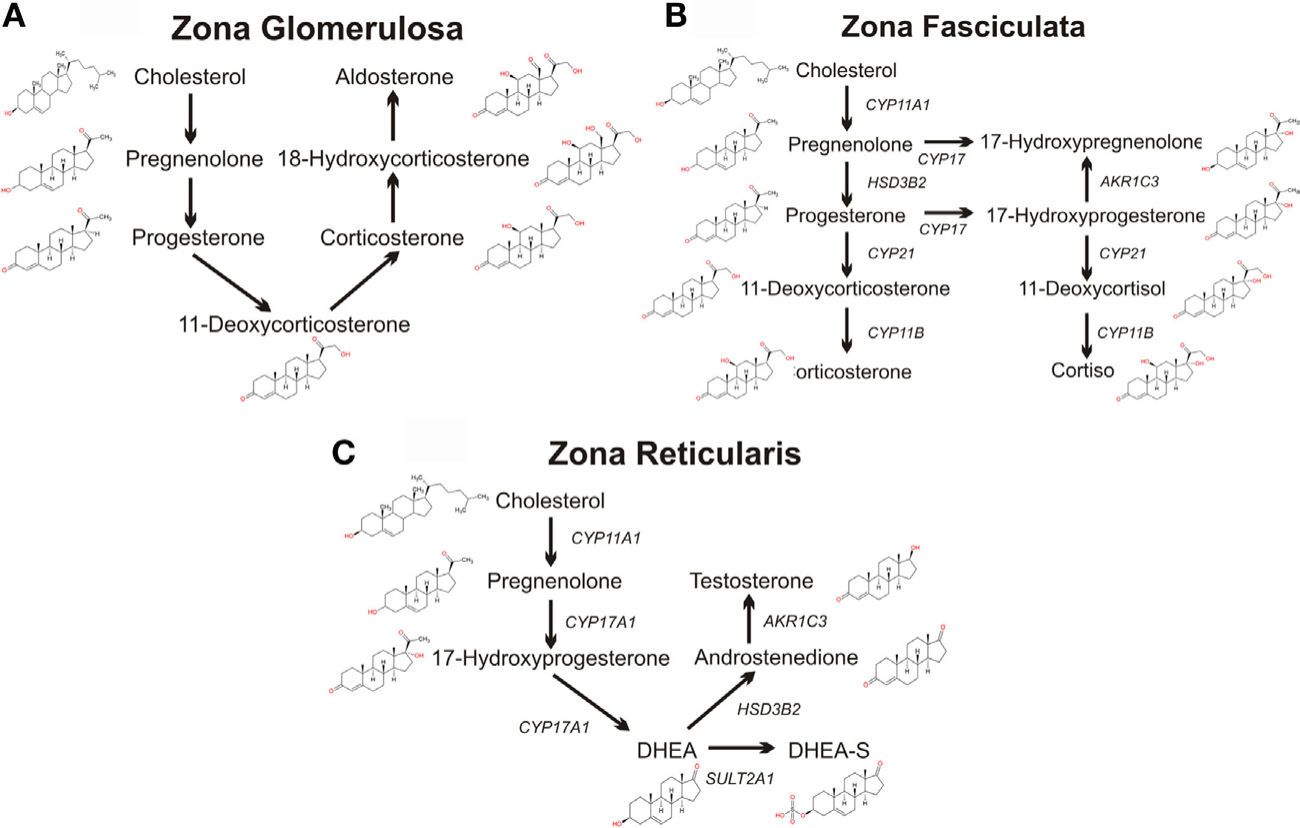
**Schematics of adrenal steroidogenic pathways**. The metabolism of cholesterol to pregnenolone by the mitochondrial CYP11A1 is common to all three zones of the human adrenal. **(A)** The mitochondrial/microsomal enzyme HSD3B converts pregnenolone to progesterone, which is metabolized to 11-deoxycorticosterone by the microsomal CYP21. The final reactions of aldosterone synthesis are catalyzed by the mitochondrial CYP11B2, which converts 11-deoxycorticosterone to corticosterone, which is hydroxylated at C18 to form 18-hydroxycorticosterone which is then finally converted to aldosterone. **(B)** In the zona fasciculata, the microsomal CYP17 and the mitochondrial/microsomal HSD3B can generate 17-hydroxyprogesterone, progesterone, and 17-hydroxyprogesterone. The microsomal CYP21 preferentially metabolizes 17-hydroxyprogesterone to 11-deoxycortisol, which is finally metabolized to the glucocorticoid cortisol by the microsomal CYP11B2. CYP21 can also metabolize progesterone to 11-deoxycorticosterone, which CYP11B2 converts to the glucocorticoid corticosterone, although this pathway is secondary in humans (although the principal pathway in rodents). **(C)** In the zona reticularis, CYP17 hydroxylates pregnenolone to 17-hydroxypregnenolone, and then DHEA. DHEA is the major steroid product of the reticularis, with sulfated DHEA (DHEA-S), androstenedione, and testosterone serving as only minor steroidogenic products.

The zona glomerulosa, lying under the adrenal capsule (Figure 1), functions as part of the renin–angiotensin–aldosterone endocrine axis and contributes to organismal electrolyte balance (6). In response to the peptide angiotensin-II, or elevated plasma potassium, the cells of the zona glomerulosa secrete aldosterone. Aldosterone, such as all steroids, is synthesized from cholesterol *via* a multienzyme pathway particular to each steroidogenic tissue, resulting in successive modifications to the sterol backbone (Figure 2). Aldosterone, a mineralocorticoid, in turn promotes sodium and water retention, as well as potassium excretion by the kidney (7). Ultrastructurally, glomerulosa cells are characteristically contain numerous mitochondria with lamelli form cristae and some lipid droplets in the cytoplasm (8). The zona fasciculata, the next layer of the adrenal cortex (Figure 1), is responsible for organismal glucocorticoid production – cortisol in humans, corticosterone in rodents (Figure 2) (3). The cells of the zona fasciculata participate in the hypothalamic–pituitary–adrenal endocrine signaling axis and respond to pituitary adrenocorticotropic hormone (ACTH) signaling through the ACTH receptor (M2CR) and its accessory protein, the melanocortin 2 receptor accessory protein (MRAP). The fasciculata cells are organized in cord-like bundles – the fascicles – surrounded by fenestrated capillaries (8). Ultrastructurally, these cells also contain numerous mitochondria, although their cristae take a more tubulovesicular form. Fasciculata cells, consistent with their prolific capacity to synthesize glucocorticoids, contain prominent smooth endoplasmic reticulum (ER) and large numbers of lipid droplets (9). The layer of the cortex abutting the medulla in humans, the zona reticularis, is not part of currently well-defined endocrine axis, but does secrete significant amounts of the androgen dehydroepiandrosterone (DHEA; Figure 2) (10). The cells of the zona reticularis resemble those of the fasciculata ultrastructurally, although contain relatively fewer lipid droplets with comparatively greater numbers of lysosomes (9).

Developmentally, the adrenal cortex arises from the adrenocortical primordium, itself derived from the urogenital ridge, a specialized region of the embryonic coelomic epithelium that also serves as the developmental precursor of the kidneys and hematopoietic progenitors (11). Cells in the adrenocortical primordium express the transcription factor genes Wilms tumor suppressor-1 (WT1), GATA-binding protein 4 (GATA4), and steroidogenicfactor-1 (SF1/NR5A1) (8, 12, 13). As development proceeds, adrenal progenitor cells in the migrate dorsomedially from the adrenocortical primordium into subjacent mesenchyme, concurrently upregulating expression of SF1, and downregulating expression of WT1 and GATA4 (13, 14). The developing adrenal gland is subsequently innervated by sympathoblasts from the neural crest, the precursors of the chromaffin cells of the medulla (15), and finally enveloped by capsule cells derived from the surrounding mesenchyme.

## Cellular Compartmentalization and Mitochondrial Respiration

Eukaryotic cells are characteristically compartmentalized, containing numerous membrane-bounded organelles, each with specialized functions. These organelles achieve their specialization through non-uniform segregation of molecules, whether they are nucleic acids, proteins, lipids, or carbohydrates. The mitochondria are famously known as the “powerhouse of the cell” for their respiratory capacity and synthesis of adenosine triphosphate (ATP). Although mitochondrial energetics have traditionally served an ancillary role in steroidogenic research (16), the recent finding that mitochondrial function directly impacts neuroendocrine, metabolic, inflammatory, and transcriptional responses to acute psychological stress (17) prompts a brief review. We will introduce individual proteins involved and use the mitochondrial respiratory chain serves an example of higher order functional protein assemblies.

The mitochondria generate ATP by oxidizing hydrogens derived from carbohydrates (through the tricyclic acid cycle) and fats (through fatty acid β-oxidation). Electrons from nicotinamide adenine dinucleotide (NADH) are donated to the iron–sulfur (Fe–S) clusters of mitochondrial complex I (NADH dehydrogenase), a multimeric inner mitochondrial membrane (IMM) protein complex. From Complex I, the electrons are sequentially shuttled to ubiquinone (coenzyme Q/CoQ), giving rise to ubiquinol (CoQH_2_). Ubiquinol transfers its electrons to a cytochrome-containing IMM protein complex, Complex III (ubiquinol/cytochrome *c* oxidoreductase), which further shuttles the electrons to cytochrome *c*. From cytochrome *c*, the electrons flow to the cytochrome-containing Complex IV (cytochrome *c* oxidase, COX), the terminal IMM protein complex of the mitochondrial respiratory chain, which uses the electrons to reduce O_2_ to yield H_2_O. The free energy of electron movement through the ETC is used to pump protons (H^+^) out of the mitochondrial matrix into the mitochondrial intermembrane space (IMS), creating a capacitance across the IMM. This potential energy is utilized to drive ATP synthesis by the IMM Complex V (ATP synthase), which condenses ADP + P_i_ to form ATP while pumping protons back into the matrix. Matrix ATP is then exchanged with cytosolic ADP by the adenine nucleotide translocator (ANT/SLC25), which works in conjunction with the outer mitochondrial membrane (OMM) voltage-dependent anion channel (VDAC) to form an energy-transducing mitochondrial contact site (18).

An interesting development in mitochondrial respiratory electron transport and one that, as discussed below, offers insight into mitochondrial steroidogenesis, began at the beginning of the twenty-first century with the proposal that the mitochondrial respiratory chain was organized in supramolecular assemblies termed “respirasomes” (19). This paradigm shift changed the view of mitochondrial electron transport from one of randomly organized respiratory chain complexes – in which components associated *via* random diffusion (20) – to one of respiratory chain supercomplexes locally transferring electrons between restricted components (21). Numerous studies utilizing blue native gel electrophoresis, preserving the fidelity of weakly associated members of protein complexes (22), have repeatedly shown that the oxidative phosphorylation complexes associate in supercomplexes of the three proton-translocating units: Complex I, Complex III, and Complex IV (19, 23, 24). Moreover, these supercomplexes have been demonstrated to be functional *in vitro*, further supporting a role for complex molecular assemblies in cellular function (25).

The conceptual justification of such supercomplexes derives from theoretical work indicating that spatial proximity of chemical reactions fosters efficiency, providing fitness advantages for evolutionary selection. This concept was originally proposed by Welch in the 1970s (26) as the concept of the “metabolon,” and subsequently popularized by Srere (27), among others. This is not a novel concept for steroidogenic research, as Lieberman and Prasad utilized the metabolon construct in their work on steroidogenic metabolism (28). The crux of the metabolon is that groups of enzymes and/or proteins within metabolic pathways physically associate. This association, as noted above, would facilitate metabolite channeling, increasing the regulation, control, and speed of metabolic pathways. The metabolon appeals to common sense and biological evolutionary arguments, as increased efficiency is a requirement of biological survival in hostile environments. However, the metabolon has been difficult to demonstrate experimentally. Biochemical pull-down experiments have been reticent to demonstrate large biological metabolic complexes (29), and it has been argued that metabolic protein complex association is weak to easily facilitate regulation. The development of non-denaturing molecular assays, such as the blue native PAGE described above, as well as high-resolution fluorescent microscopy, has supported research into transient, but functionally critical, aspects of protein–protein interactions. Indeed, increasing evidence of such interactions in cellular metabolism is receiving attention, ranging from descriptions of cytoplasmic purinosomes – regulating purine biosynthesis (30, 31) – to mitochondrial fatty acid translocation machinery – contributing to mitochondrial fatty acid beta-oxidation and energetics (32, 33). The following sections describe the molecular components of mitochondrial steroidogenic machinery, first introducing individual proteins before transitioning description of a model of steroidogenesis functionally incorporating these proteins into larger protein assemblies, much akin to what has been observed for mitochondrial respiration.

## Steroidogenic Cells, Proteins, and Mitochondria

Cholesterol serves as the metabolic precursor of all adrenal steroids, and as such, the adrenal cortex can be viewed as a highly specialized lipid processing organ. Cholesterol possesses fascinating structural characteristics, making it one of the most studied and versatile molecules in biological systems. Steroid hormones, in turn, are oxygenated forms of cholesterol, a characteristic they share with bile acids and oxysterols (2), and one which changes their chemical properties from highly hydrophobic to modestly hydrophilic. This change in chemical properties underlies the shift in their biological role from structural (as cholesterol plays in lipid membranes) to informational (as steroids play in biological signaling).

Any discussion of the steroidogenic mitochondria of the adrenal gland must revolve around the mitochondrial steroidogenic enzymes. These steroidogenic enzymes fall into two broad families: the cytochrome P450 (CYP) and hydroxysteroid dehydrogenase/ketosteroid reductase (HSD/KSR) enzymes. The CYP enzymes represent the majority of the steroidogenic enzymes and will receive the greatest attention here as they are the most abundant steroidogenic enzymes of mitochondria. A highly diverse superfamily of enzymes, the CYPs are characterized by a single heme prosthetic group and the ability to absorb light at 450 nm when reduced with carbon monoxide. Enzymatically, P450 enzymes exhibit an extraordinary ability to insert oxygen into non-activated carbon–hydrogen bonds at the same time exhibiting high structural selectively. They accomplish this feat through their ability to activate molecular oxygen, although the iron atom of their heme tetrapyrrole prosthetic group with the aid of an ancillary redox partner (34).

**Figure 3:**
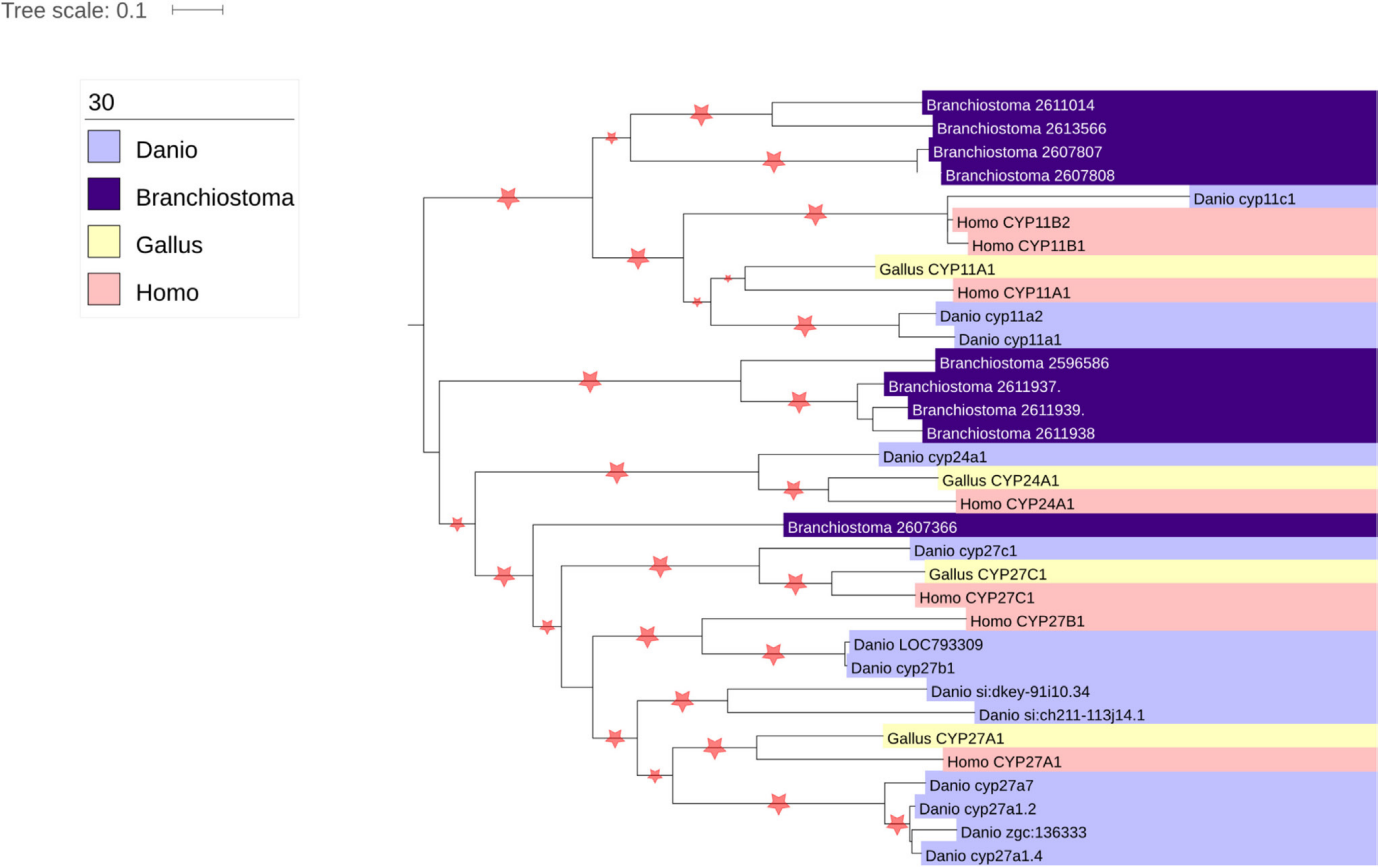
**Phylogenetic analysis of vertebrate mitochondrial CYP evolution**. Homologs of the seven human mitochondrial CYPs (CYP11A1, CYP11B2, CYP11B3, CYP24A1, CYP27A1, CYP27B1, and CYP27C1) were identified using the nucleotide BLAST tool (41). Organisms chosen for search included the fully sequenced chicken (*Gallus gallus*), zebrafish (*Danio rerio*), and lancelet (*Branchiostoma floridae*), as they represented divergent vertebrate sequences (chicken vs. human vs. zebrafish) that could be compared with a non-vertebrate chordate (lancelet). The genomes of the Western clawed frog (*Xenopus tropicalis*) and anole lizard (*Anolis carolinesis*) were also searched, but sequences from these organisms were not included in the analysis as surprisingly no homologs of CYP11B1 or CYP11B2 were found (*data not shown*). Nucleotide sequences were aligned using MUSCLE (42), and phylogenetic trees constructed using PHyML (43).

Thousands of CYPs have been identified, in all domains of life, suggesting that this class of protein has ancient roots. There are 57 CYP genes encoding CYP enzymes in humans, subdivided into 18 clades. The majority of the CYP enzymes are localized to the ER, but one clade is localized to the mitochondria. While ER-localized CYPs obtain their electrons from a single P450 oxidoreductase, the mitochondrial CYPs utilize an electron transport chain containing the ferredoxin reductase (FDXR) and ferredoxin proteins (34). There are seven mitochondrial CYPs in humans, namely CYP11A1, CYP11B1, CYP11B2, CYP24A1, CYP27A1, CYP27B1, and CYP27C1. Of these, CYP11A1, CYP11B1, and CYP11B2 are involved in steroidogenesis, metabolizing cholesterol and steroids. Interestingly, CYP24A1 and the three CYP27 isozymes are all involved in cholesterol metabolism, playing roles in bile acid, oxysterol, and vitamin D biosynthesis (35, 36), suggestive that the ancestor of the mitochondrial clade of enzymes was involved in sterol metabolism. Interestingly, however, phylogenomic analysis of the CYP enzymes has repeatedly shown that the CYP11 family appears with, or shortly before, the emergence of vertebrates (37, 38) (Figure 3). Indeed, it has been postulated that the advent of steroidogenesis and steroid hormone receptor signaling and the increased developmental complexity of vertebrates are intimately linked (39). Moreover, evolution of the mitochondrial CYP11 family is itself of interest within the vertebrate lineage, for while homologs of the CYP11 family, responsible for the first step of synthesis for all steroids, are observed throughout vertebrata from fish to mammals (37, 38) (Figure 3), the CYP11B family, responsible for glucocorticoid and mineralocorticoid synthesis, is underrepresented throughout this subphylum (Figure 3). Thus, while the molecular details of adrenal mitochondrial steroid biosynthesis are well understood, as described below, the evolution of this system remains an important and poorly understood area of research (7, 40).

## Cholesterol Side Chain Cleavage: CYP11A1

CYPA11A1 is absolutely essential for the synthesis of all vertebrate steroids, which are all characterized by the CYP11A1 reaction, namely cleavage of the cholesterol aliphatic side chain. The proposed reaction mechanism of CYP11A1 involves three sequential modifications of cholesterol. In the first step, CYP11A1 hydroxylates cholesterol at carbon 22 of the aliphatic tail; in the second step, cholesterol is hydroxylated at the carbon 20 of the aliphatic tail; finally, in the third step, oxidative scission of the C20–22 bond of the subsequent 20,22-dihydroxycholesterol yields the steroid pregnenolone and the reactive aldehyde, isocaproaldehyde (44). This reaction mechanism, originally proposed in the 1970s based on purified enzyme catalysis of hydroxycholesterol (45), has found support in the publishing of x-ray crystal structures of bovine and human CYP11A1 (46, 47), which indicate that the heme prosthetic group lies proximally to the 20′ and 22′ carbons of cholesterol. Recent high-resolution temporal enzymatic work further indicates that transient cholesterol hydroperoxyl serves as reaction intermediates (48, 49), further supporting the sequential oxidative cleavage model of this enzyme.

The cellular expression CYP11A1 is hormonally regulated in the steroidogenic tissues of the adrenal and gonads, with circulating pituitary hormones stimulating intracellular cAMP production, which in turn promotes CYP11A1 expression (50). A number of paracrine and endocrine factors affect the expression of CYP11A1. Pituitary hormones, such as ACTH or angiotensin-II, stimulate CYP11A1 expression through a cAMP-dependent mechanism (51, 52), and the human CYP11A1 promoter contains two cAMP-responsive regions (53). Additional factors that stimulate cAMP in adrenocortical cells, such as activin (54), are also able to stimulate CYP11A1 expression. In contrast to cAMP, intracellular signaling pathways activated by Ca^2+^ and protein kinase C (PKC) can suppress CYP11A1 transactivation (55). In addition to the cAMP-responsive sites (CRSs) in the CYP11A1 promoter, the transcription factor SF-1 also contains an activating site (56, 57), which modulates the basal and cAMP-stimulated levels of CYP11A1 expression through association with the transcription factors p300 and CREB-binding protein (CBP) (58).

## Glucocorticoid and Mineralocorticoid Synthesis: CYP11B1 and CYP11B2

The final steps in the synthesis of glucocorticoids and mineralocorticoids are catalyzed by two closely related mitochondrial enzymes: CYP11B1 and CYP11B2 (59). Similar to CYP11A1 and the other mitochondrial CYPs, CYP11B1 and CYP11B2 are associated with the IMM and are expressed with leader peptides targeting them to the mitochondrial matrix (60). CYP11B1 is the more abundantly expressed of the two proteins and is expressed predominantly in the zona fasciculata, and to a lesser extent in the zona reticularis, but not in the zona glomerulosa (61). CYP11B1 catalyzes the 11β-hydroxylation of 11-deoxycorticosterone and 11-deoxycortisol yielding corticosterone and cortisol, respectively (Figure 2) (44). CYP11B1 also has the capacity to hydroxylate C18 of 11-deoxycorticosterone or corticosterone to form 18-hydroxycorticosterone (62); however, it cannot catalyze the oxidation of the 18-hydroxy group to form aldosterone. This last reaction is catalyzed by CYP11B2, which is able to catalyze the sequential 11β-hydroxylation of 11-deoxycorticosterone, the hydroxylation of C18 and the subsequent oxidation of C18 to yield the C18 aldehyde group of aldosterone (Figure 2) (63–65). CYP11B2 inefficiently catalyzes oxidation of corticosterone; this finding has lent support to the zonation theory of mineralocorticoid and glucocorticoid synthesis, as the products of CYP11B1 would not be sequentially catalyzed to aldosterone (65).

Although it is unclear whether the CYP11B clade of mitochondrial CYPs emerged with the advent of vertebrates or mammals, within the mammalian lineage, the CYP11B clade exhibits significant diversity. While humans possess the two enzymes discussed above, cattle and pigs possess a single enzyme, CYP11B (66, 67), which is able to catalyze all of the reactions of CYP11B1 and CYP11B2. Conversely, within the rodent lineage, rats (but not mice) possess three CYP11B genes – CYP11B1, CYP11B2, and CYP11B3 – with CYP11B1 and B2 exhibiting homologous activity to their human orthologs, and CYP11B3 possessing the ability to convert deoxycorticosterone to 18-deoxycorticosterone, but lacking the 18 activity necessary to synthesize aldosterone (68).

CYP11B1 and CYP11B2 are located on human chromosome 8q21–22 (59, 69). Consistent with a tandem duplication event, the two human genes are closely linked, separated by 40 kb, exhibiting similar intron/exon structure, and 90 and 95% identity in the coding and non-coding sequences, respectively (59, 70). CYP11B1 expression is induced by ACTH *via* cAMP (71, 72), through a mechanism relying on cAMP response element (CRE) and activating transcription factor (ATF) sequences in the CYP11B1 promoter (73). Orphan nuclear receptors play a critical role in CYP11B1 genetics, with SF1/NR5A1 and LRH-1/NR5A2 both contributing to the relative expression of CYP11B1 and regulating the comparative expression of CYP11B1 vs. CYP11B2 (73, 74). CYP11B2 transcription in granulosa cells is induced by potassium and by angiotensin-II, in both cases stimulating calcium (Ca^2+^) influx and stimulation of PKC and calmodulin-dependent protein kinase kinase (CAMKK) intracellular signaling (75, 76). CYP11B2 expression requires the action of the transcription factors NURR1 and NGF1B, but interestingly contrasts with CYP11B1 in its relationship with the transcription factor SF-1 (77, 78). Transcriptional regulation of CYP11B2 is also influenced by the activity of chicken ovalbumin upstream promoter transcription factor I (COUP-TF1), which itself is coactivated by the small ubiquitin-related modifier-1 (SUMO-1) conjugase and ligase Ubc9 and PIAS1 (79).

## Electron Transfer to CYP11A1: Ferredoxin Reductase and Ferredoxin

**Figure 4:**
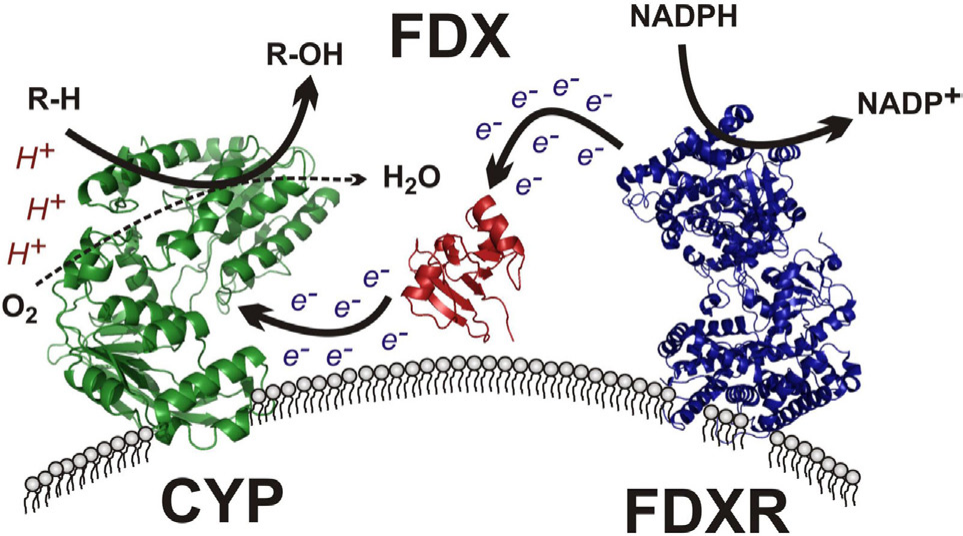
**Mitochondrial cytochrome P450 electron transport chain**. The membrane-bound flavin-containing ferredoxin reductase (FDXR) accepts two electrons from NADPH, yielding NADP^+^. These electrons are passed to the iron–sulfur cluster of ferredoxin (FDX), which donates the electrons to the heme prosthetic group of the mitochondrial cytochrome P450 (CYP), which uses protons and molecular oxygen to hydroxylate its target substrate (R–H) yielding the final hydroxylated product (R–OH) and water.

The mitochondrial clade of CYP enzymes uses two sequential electron-transfer donors – ferredoxin (FDX) and FDXR – as intermediates in electron donation from NADPH (Figure 4). These are ancient proteins, with orthologs expressed in all domains of life and involved in numerous processes outside of steroidogenesis (80). FDXR is a 54.5-kDa flavoprotein affixed to the IMM that reduces NADPH and contains of two domains (81): (1) a NADPH-binding domain and (2) a flavin adenine dinucleotide-binding domain, with electron transfer occurring between the two. This cleft possesses a number of basic residues, residues essential for interaction with acidic residues on its electron donor partner, FDX (82). FDXR is broadly expressed in numerous tissues, but is comparatively abundant in steroidogenic tissues (83), where its abundance contributes to the catalytic activity of the mitochondrial CYPs (84). High SF-1 expression in steroidogenic tissue likely contributes this preponderance of FDXR, as SF-1-binding sites are present in the FDXR promoter and SF-1 overexpression in adrenal cell models drives FDXR expression (85).

The electron donor partner of FDXR, FDX, is a 14-kDa mitochondrial matrix-localized protein containing a Fe–S cluster tethered by four cysteine residues (86). Two ferredoxins exist in humans, with FDX1 supporting steroidogenesis and FDX2 participating in heme and Fe/S cluster protein synthesis (87). After obtaining electrons from FDXR, FDX1 subsequently transfers its electrons to mitochondrial P450 enzymes, including CYP11A1, CYP11B1, and CYP11B2, among others (Figure 4). FDX has been described as a mobile, indiscriminate, diffusible electron shuttle (3), much as cytochrome *c* and ubiquinol have been described previously (20).

## Identification of Steroidogenic Protein Complexes

**Figure 5:**
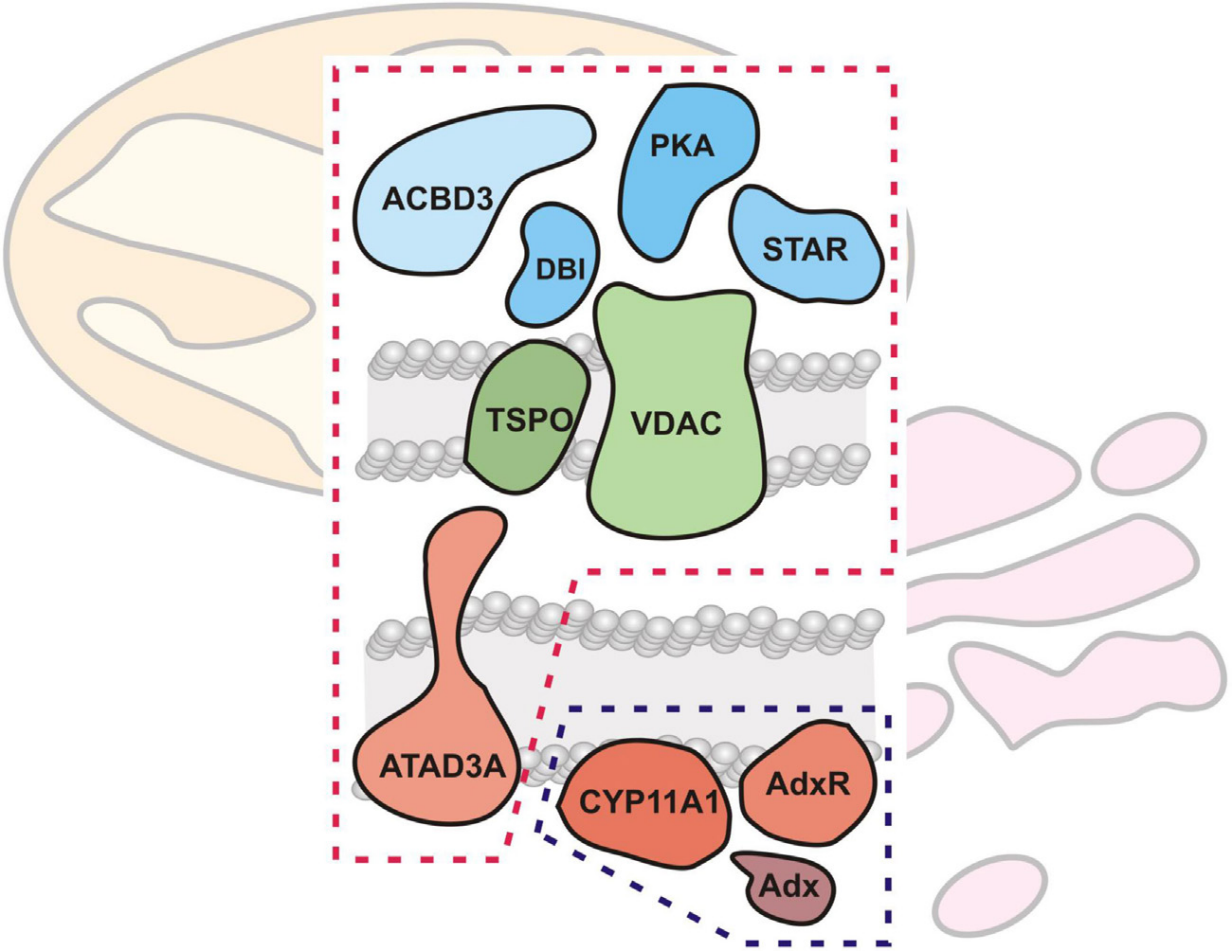
**Mitochondrial cholesterol transport and metabolism machinery**. The mitochondrial cholesterol import and metabolism machinery are shown, demarcated by red (transduceosome) and blue (metabolon) dashed lines, respectively. The transduceosome contains cytoplasmic (StAR, ACBD3, DBI, and PRKARI; colored blue), OMM (VDAC and TSPO; colored green), and IMM (ATAD3A; colored red) components which assemble in response to hormonal stimulation and transduce the resultant cAMP signal to the mitochondria for cholesterol import. It is important to note, however, that molecular details of cholesterol import are still lacking. Once cholesterol is imported into the mitochondria, the IMM metabolon (CYP11A1, FDX, and FDXR; colored red) metabolizes cholesterol to pregnenolone, the precursor to all other steroids, including adrenal glucocorticoids and mineralocorticoids.

Research on CYP enzymes has contributed to the emerging picture of CYPs operating in functional complexes with other CYPs (88) as well as with their cognate electron donor partners (89). As noted in the previous section, the mitochondrial CYPs and the FDX and FDXR redox partners have been considered to interact randomly in the mitochondrial inner membrane (3). However, examination of native protein complexes in steroidogenic mitochondria from tumor Leydig cells using BN-PAGE and mass spectroscopy suggested that CYP11A1 and FDXR physically associate (90). In addition, these natively isolated CYP11A1 complexes were functionally active, cleaving the aliphatic tail of a fluorescent cholesterol reporter. Taken together, these findings support a model for CYP11A1–FDXR electron transport occurring in a physically associated metabolon, much akin to the electron transport of the respirasomes discussed above (24). This CYP11A1 metabolon model integrates with work on the steroidogenic transduceosome, a multiprotein complex traversing the OMM and IMM of the steroidogenic mitochondria integrating the movement of cholesterol with intracellular signaling to CYP111A1 (90, 91) (Figure 5). The steroidogenic transduceosome and metabolon complexes contain a number of cytoplasmic and mitochondrial components (Figure 5): cytoplasmic proteins include the steroidogenic acute regulatory (StAR) protein, the protein kinase cAMP-dependent type I regulatory subunit alpha (PRKAR1A), the diazepam-binding inhibitor (DBI), and the acyl-CoA-binding domain containing 3 (ACBD3); mitochondrial proteins include the VDAC, the translocator protein (TSPO), ATPase family, AAA domain containing 3A (ATAD3A), CYP family 11 subfamily A member 1 (CYP11A1), and FDXR. In addition, functional partners of the transduceosome have been identified, including mitochondria-associated members of the 14-3-3 adaptor protein family (92, 93) as well as kinase signalers such as extracellular signal-regulated kinase (ERK) 1/2 (94); the physical association and temporal interaction of these proteins with the transduceosome remain an active area of research. Because the focus of this review is upon mitochondrial contributions to steroidogenesis, the mitochondrial components of the transduceosome and metabolon are focused upon below.

## Voltage-Dependent Anion Channel

Voltage-dependent anion channel is the most abundant protein of the mitochondrial outer membrane and is widely accepted as the principal route and control of metabolic flux between the cytosol and mitochondria (95). VDAC is a 32-kDa beta barrel protein and has been implicated in numerous cellular processes, ranging from cellular energetics to apoptosis (96). Three VDAC isoforms are expressed in the human genome, with VDAC1 located on chromosome 5q31, VDAC2 located on chromosome 10q22, and VDAC3 located on chromosome 8p11 (97). The relative abundance of the different VDAC isoforms vary by tissue, by VDAC1 is the predominant form, followed by VDAC2, with VDAC3 expression low in comparison. Although functional redundancy is believed to exist between the isoforms, significant differences in roles of the VDAC proteins has been slowly teased out through biochemical and genetic investigations (98). Interestingly, mice null for *Vdac1* and *Vdac3* are viable and display little overt phenotypical changes; however, on this particular murine genetic background, *Vdac2*^−/−^ mice were embryonically lethal (99, 100). Moreover, although the VDAC proteins have been recurrently implicated in apoptosis, mitochondria from *Vdac1*^−/−^ and *Vdac3*^−/−^ null mice, as well as cell lines null for all three isozymes fail to show changes in mitochondrial permeability transition and Bcl-2 family member-driven cell death compared with wild-type mitochondria.

Voltage-dependent anion channel is found at contact sites between the OMM and the IMM (101) where it may complex with energetics-related proteins, such as hexokinase, ANT, and creatine kinase, or with apoptotic proteins of the Bcl-2 family (18), and as noted above, and discussed below in greater detail, appears to play a central role in facilitating mitochondrial steroidogenic cholesterol transport through interactions with several key proteins. Moreover, VDAC also appears to play a significant, albeit poorly understood, role in cellular cholesterol homeostasis (102). Structural characterization of VDAC indicates that it is a cholesterol-binding protein; whether this cholesterol binding is a non-specific artifact of VDAC hydrophobicity, or whether this cholesterol binding plays a physiological role in steroidogenesis or other processes remains to be determined. It is unlikely that VDAC itself participates as a cholesterol channel, as the center of its ring-like structure is hydrophilic, suitable for anion transport but unsuitable for hydrophobic molecule transport (103, 104). After nearly half a century of work on this ubiquitous protein, much remains to be understood, especially in the context of mitochondrial cholesterol metabolism.

## Translocator Protein, 18 kDa

In the 1970s and 1980s, studies of benzodiazepine drug binding to sites outside of the central nervous system led to the identification of a peripheral benzodiazepine-binding site, generally expressed throughout the body, but concentrated in the steroidogenic cells of the adrenal and gonad (105–107). Isolation of this benzodiazepine-binding site led to the identification of an 18-kDa integral OMM protein, originally named the peripheral benzodiazepine receptor (PBR) (106). Subsequent research demonstrated that benzodiazepine and other chemical distinct PBR ligands were able to stimulate steroid biosynthesis by mobilizing mitochondrial cholesterol transport in isolated mitochondria, cell cultures, as well as in humans (108–111), and based on work implying the involvement of this protein in mitochondrial cholesterol and heme tetrapyrrole import, the PBR was renamed the TSPO (112).

Biochemical evidence and recent structural determinations of the TSPO protein indicate that TSPO is predominantly α-helical, containing five helices clustered into a barrel-like shape (113–115). Although antioxidant properties of TSPO have been demonstrated (115), no classical redox enzymatic or prosthetic groups, such as transition metals or active thiols, have been observed, and functional analysis of the protein has been difficult owing to its hydrophobicity (116). TSPO does contain an evolutionarily conserved C-terminal cholesterol recognition amino acid concensus (CRAC) motif (117, 118), which has been shown to facilitate cholesterol binding through a conserved tyrosine residue (119). This cholesterol-binding activity for TSPO has been implicated in steroidogenesis, as small molecules targeting the CRAC motif inhibit steroid production in cell and animal models (120, 121), and a naturally occurring human polymorphism in the protein proximal to the C-terminal CRAC motif (A147T) reduces cellular steroid production (122).

Hormonal stimulation of steroidogenesis in a steroidogenic cell model resulted in increased TSPO polymerization in correlation with increased ligand-binding affinity and steroid production (123, 124). Much like VDAC discussed above, TSPO has been identified as concentrated at OMM–IMM contact sites (125), and in addition to homooligomerization, TSPO has been consistently shown to physically associate with VDAC (126–128). Moreover, VDAC–TSPO interaction affects binding of TSPO ligands (127), suggestive of a functional relationship between the two proteins. The TSPO–VDAC platform appears to serve as an OMM base for the steroidogenic transduceosome cholesterol-transfer machinery (90, 91), a complex which appears to predominantly contain the polymerized form of TSPO (90). However, the mechanistic details of TSPO involvement in this complex are unclear at this time. Recent genetic mouse models in which TSPO had been deleted tissue specifically and globally have yielded conflicting results regarding steroidogenesis, ranging from no effect on steroidogenesis to severe compromise of ACTH-stimulated production of corticosterone (129–131). The complex effects of genetic background and selection for compensatory changes likely play a role in the experimental variability, however, especially in light of skewed embryonic Mendelian ratios in TSPO null mice (131).

## Steroidogenic Acute Regulatory Protein

Pharmacological inhibition of protein synthesis by compounds, such as cycloheximide, was long known as an inhibitor of steroidogenesis (132), suggesting that rapid protein synthesis was a necessary driver of steroid biosynthesis. The StAR protein was originally identified as a labile protein factor rapidly induced in response to hormonal stimulation of steroidogenic cells in correlation with increased steroid production (133, 134). Moreover, a number of studies have demonstrated that StAR is a direct target of hormonally stimulated cellular kinase signaling pathways, including the protein kinase A (PKA) and ERK kinase pathways (94, 135, 136). The necessity of StAR for steroidogenesis derived from work in humans showing that a broad spectrum of mutations in StAR contribute to congenital adrenal lipoid hyperplasia, a condition characterized by the inability to synthesize steroids, resulting in impaired sexual development and adrenal dysfunction leading to infant death unless treated with glucocorticoid supplementation (137).

Steroidogenic acute regulatory protein is located on chromosome 8p11 and is expressed as a 37-kDa mitochondrial pre-protein containing a mitochondrial targeting leader sequence, and subsequently imported into mitochondria, where the presequence is cleaved by Lon proteases in the matrix to a 30-kDa mature protein (although it is interesting to note that theoretical calculation of StAR protein molecular mass indicate 30 and 25 kDa masses for the pre- and mature proteins, although the reason for this discrepancy is unclear at this time). Surprisingly, StAR was found to stimulate steroid production in cell model systems without its leader sequence and import into the mitochondria (138). This led to elegant work on StAR action involving its molecular tether to the OMM, IMS, and IMM which indicated that StAR acts on the OMM and that mitochondrial import to the matrix inactivates StAR activity (139). Interestingly, however, *in vivo* work has demonstrated that the StAR mitochondrial presequence has biological necessity, as mouse models in which full-length StAR has been replaced by StAR that lacks the mitochondrial targeting sequence stochastically exhibit the CAH phenotype of steroidogenic failure (140). Moreover, CAH-causing mutations have been found in the leader sequence of human StAR-mutation patients (141), collectively arguing that the StAR relationship with its leader peptide is more complex than previously thought. The finding that StAR physically interacts with VDAC1 (142) and can be found in the OMM transduceosome complex when proteins are crosslinked after steroidogenic stimulation (91) supports a model in which StAR serves as a “key” acting to “start” the mitochondrial cholesterol import machinery. However, the precise molecular details of this model remain to be determined.

## ATPase Family, AAA Domain Containing 3A

The ATAD3A protein belongs to the AAA^+^ family of ATPases, a broadly conserved family of ATPases implicated in various cellular processes (1, 3–5). ATAD3A is characterized by two N-terminal coiled-coil domains: a central transmembrane helix and a conserved C-terminal AAA^+^-type ATPase domain (143). Several studies have localized ATAD3A to the mitochondria (143, 144), where is appears to be involved in mitochondrial membrane dynamics. Trypsin digestion assays have been used to study the membrane topology of ATAD3A, suggesting that the C-terminal ATPase domain is localized in the mitochondrial matrix, the transmembrane segment traverses the IMM, and the N-terminal coiled-coils anchor the protein the OMM. ATAD3A appears capable of homooligomerization, and as noted above, appears to be a partner protein in the mitochondrial transduceosome of steroidogenic cells, critical for cholesterol import into mitochondria and steroidogenesis (90, 145).

## Mitochondria-Associated Membranes: Sites of ER–Mitochondrial Membrane Interaction

The ER is a complex cellular organelle, formed by an interconnected network of cisternae (146), is distributed across the cell and is involved in numerous processes, including lipid and protein synthesis. The ER is well known in steroidogenic research as the site of action of numerous steroidogenic CYP and HSD/KSR enzymes (3), but recently, the ER has attracted recent interest in mitochondrial cholesterol metabolism as a possible source of cholesterol (147). The ER and mitochondria are considered cholesterol poor organelles, especially in contrast to the high levels present in plasma membranes and endosomes. However, the ER appears to be a staging platform for cellular cholesterol homeostasis, as endogenous cellular cholesterol is synthesized in the ER, and cholesterol taken up by cells from circulating lipoproteins makes its way to the ER before incorporation into lipid droplets. In addition, the ER houses the sterol regulatory element-binding protein (SREBP) sensory machinery, which senses ER sterol levels and subsequently regulates transcription of genes involved in cholesterol and fatty acid synthesis and uptake (148). Although lipid droplets have been considered the classic source of steroidogenic cholesterol, the intimate relationship between lipid droplets, the ER and mitochondria, suggest a complex relationship in mitochondrial cholesterol delivery for steroidogenesis (149).

Almost 30 years ago, Vance demonstrated phospholipid synthesis in cellular fractions enriched in mitochondrial and ER markers (150). Electron microscopic investigations of mitochondria–ER association have consistently revealed the existence of specific regions of close apposition between the ER membranes and the OMM, with these regions representing between 5 and 20% of the mitochondrial surface (151–153). These mitochondria-associated membrane (MAM) sites have become recognized as possessing their own particular makeup, characterized by a number of resident proteins (154). Interestingly, several of these proteins have been demonstrated to participate in mitochondrial cholesterol transport and steroid biosynthesis. VDAC itself has been demonstrated to be present in MAMs (155), and, interestingly, StAR appears to interact with VDAC2 in steroidogenic cell model MAMs, an interaction necessary for its steroidogenic activity and mitochondrial import (156). In addition to VDAC, which is predominantly localized to mitochondria, several highly enriched resident MAM proteins have been demonstrated to play a key role in mitochondrial cholesterol transport. The first of these proteins, the sigma-1 receptor (SIGMAR1), was found to coimmunoprecipitate with VDAC2 in a steroidogenic cell model as well as disrupt mitochondrial cholesterol metabolism when its expression was reduced by short interfering RNA (siRNA) (157). Interestingly, SIGMAR1 appears to promote the compartmentalization of cholesterol in ER membranes (158), although depletion of cholesterol promoted mitochondrial–ER association in *in vitro* membrane association assays and cell models (159). A second resident MAM protein, acyl-CoA synthetase 4 (ACSL4), an enzyme involved in cellular arachidonic acid metabolism, participates in mitochondrial arachidonic acid movement (160). Of note, silencing the expression of ACSL4 inhibits steroidogenesis in a cell model, overexpression of ACSL4 promotes steroidogenesis (161), and cAMP signaling promotes increased mitochondrial colocalization of ACSL4 (162), collectively suggestive of a MAM relationship to mitochondrial arachidonic acid and cholesterol import. Finally, ATAD3A, in addition to forming a physical link between the IMM and OMM, may be involved in linking the mitochondria to the ER at MAMs. A long isoform of ATAD3A was found to be present in the MAMs of steroidogenic cells (145), and this work, in conjunction with the VDAC–StAR–MAM work cited above (156), suggesting that the transduceosome complex may serve to link not only the membranes of the mitochondria to the CYP metabolon but also the metabolon to cholesterol reserves in the ER and beyond.

## Conclusion

Steroidogenesis begins with the mobilization and movement of cholesterol from intracellular stores into mitochondria. Control of steroidogenic output is organized at two levels: substrate availability and targeting, and enzyme expression and localization. Past and recent studies in hormone-inducible steroidogenic cells showed that cholesterol trafficking and targeting into mitochondria is rate limiting and driven by intracellular protein networks, referred to as the transduceosome, which amplifies the cAMP signal at the OMM, and the steroidogenic metabolon. This mitochondrial metabolon prevents unwanted crosstalk of the substrate cholesterol with other pathways, optimizing substrate concentration and targeting to CYP11A1. Deciphering the organization and regulation of intracellular protein assemblies that interact with the steroidogenic machinery will provide insight into the intracellular events involved in normal and disease states, facilitating diagnosis and treatment. These studies suggest a shift in focus in steroidogenic cell biology from the actions of individual proteins in isolation to the actions of protein assemblies working together to execute specialized cellular functions, in this case adrenal steroid formation.

## Author Contributions

All authors listed have made substantial, direct, and intellectual contribution to the work and approved it for publication.

## Conflict of Interest Statement

The authors declare that the research was conducted in the absence of any commercial or financial relationships that could be construed as a potential conflict of interest.

## References

[1] Vinson G The adrenal cortex and life. Mol Cell Endocrinol (2009) 300(1–2):2–6. 10.1016/j.mce.2008.09.008 18840500

[2] Midzak A Papadopoulos V. Binding domain-driven intracellular trafficking of sterols for synthesis of steroid hormones, bile acids and oxysterols. Traffic (2014) 15(9):895–914. 10.1111/tra.12177 24890942

[3] Miller W Auchus R. The molecular biology, biochemistry, and physiology of human steroidogenesis and its disorders. Endocr Rev (2011) 32(1):81–151. 10.1210/er.2010-0013 21051590 PMC3365799

[4] Kvetnansky R Sabban E Palkovits M. Catecholaminergic systems in stress: structural and molecular genetic approaches. Physiol Rev (2009) 89(2):535–606. 10.1152/physrev.00042.2006 19342614

[5] Miller W. A brief history of adrenal research: steroidogenesis – the soul of the adrenal. Mol Cell Endocrinol (2013) 371(1–2):5–14. 10.1016/j.mce.2012.10.023 23123735

[6] Spät A Hunyady L. Control of aldosterone secretion: a model for convergence in cellular signaling pathways. Physiol Rev (2004) 84(2):489–539. 10.1152/physrev.00030.2003 15044681

[7] Rossier B Baker M Studer R. Epithelial sodium transport and its control by aldosterone: the story of our internal environment revisited. Physiol Rev (2015) 95(1):297–340. 10.1152/physrev.00011.2014 25540145

[8] Pihlajoki M Dörner J Cochran RS Heikinheimo M Wilson DB. Adrenocortical zonation, renewal, and remodeling. Front Endocrinol (2015) 5(6):27. 10.3389/fendo.2015.00027 25798129 PMC4350438

[9] Nussdorfer G Cytophysiology of the adrenal cortex. Int Rev Cytol (1986) 98:1–405. 3512469

[10] Warner M Gustafsson J DHEA – a precursor of ERβ ligands. J Steroid Biochem Mol Biol (2015) 145:245–7. 10.1016/j.jsbmb.2014.08.003 25125389

[11] Ariza L Carmona R Cañete A Cano E Muñoz-Chápuli R. Coelomic epithelium-derived cells in visceral morphogenesis. Dev Dyn (2016) 245(3):307–22. 10.1002/dvdy.24373 26638186

[12] Yates R Katugampola H Cavlan D Cogger K Meimaridou E Hughes C Adrenocortical development, maintenance, and disease. Curr Top Dev Biol (2013) 106:239–312. 10.1016/B978-0-12-416021-7.00007-9 24290352

[13] Bandiera R Vidal VP Motamedi FJ Clarkson M Sahut-Barnola I von Gise A WT1 maintains adrenal-gonadal primordium identity and marks a population of AGP-like progenitors within the adrenal gland. Dev Cell (2013) 27(1):5–18. 10.1016/j.devcel.2013.09.003 24135228 PMC4032791

[14] Val P Martinez-Barbera J Swain A. Adrenal development is initiated by Cited2 and Wt1 through modulation of Sf-1 dosage. Development (2007) 134(12):2349–58. 10.1242/dev.004390 17537799

[15] Lumb R Schwarz Q. Sympathoadrenal neural crest cells: the known, unknown and forgotten? Dev Growth Differ (2015) 57(2):146–57. 10.1111/dgd.12189 25581786

[16] Midzak AS Chen H Aon MA Papadopoulos V Zirkin BR. ATP synthesis, mitochondrial function, and steroid biosynthesis in rodent primary and tumor Leydig cells. Biol Reprod (2011) 84(5):976–85. 10.1095/biolreprod.110.087460 21228212 PMC3080423

[17] Picard M McManus MJ Gray JD Nasca C Moffat C Kopinski PK Mitochondrial functions modulate neuroendocrine, metabolic, inflammatory, and transcriptional responses to acute psychological stress. Proc Natl Acad Sci U S A (2015) 112(48):E6614–23. 10.1073/pnas.1515733112 26627253 PMC4672794

[18] Brdiczka D Zorov D Sheu S. Mitochondrial contact sites: their role in energy metabolism and apoptosis. Biochim Biophys Acta (2006) 1762(2):148–63. 10.1016/j.bbadis.2005.09.007 16324828

[19] Schägger H Pfeiffer K. Supercomplexes in the respiratory chains of yeast and mammalian mitochondria. EMBO J (2000) 19(8):1777–83. 10.1093/emboj/19.8.1777 10775262 PMC302020

[20] Hackenbrock C Chazotte B Gupte S. The random collision model and a critical assessment of diffusion and collision in mitochondrial electron transport. J Bioenerg Biomembr (1986) 18(5):331–68. 10.1007/BF00743010 3021714

[21] Wittig I Carrozzo R Santorelli FM Schägger H. Supercomplexes and subcomplexes of mitochondrial oxidative phosphorylation. Biochim Biophys Acta (2006) 1757(9–10):1066–72. 10.1016/j.bbabio.2006.05.006 16782043

[22] Wittig I Schägger H. Features and applications of blue-native and clear-native electrophoresis. Proteomics (2008) 8(19):3974–90. 10.1002/pmic.200800017 18763698

[23] Lenaz G Genova M. Kinetics of integrated electron transfer in the mitochondrial respiratory chain: random collisions vs. solid state electron channeling. Am J Physiol Cell Physiol (2007) 292(4):C1221–39. 10.1152/ajpcell.00263.2006 17035300

[24] Genova M Lenaz G. Functional role of mitochondrial respiratory supercomplexes. Biochim Biophys Acta (2014) 1837(4):427–43. 10.1016/j.bbabio.2013.11.002 24246637

[25] Acín-Pérez R Fernández-Silva P Peleato ML Pérez-Martos A Enriquez JA. Respiratory active mitochondrial supercomplexes. Mol Cell (2008) 32(4):529–39. 10.1016/j.molcel.2008.10.021 19026783

[26] Welch G On the role of organized multienzyme systems in cellular metabolism: a general synthesis. Prog Biophys Mol Biol (1977) 32(2):103–91. 10.1016/0079-6107(78)90019-6 143673

[27] Srere P Complexes of sequential metabolic enzymes. Annu Rev Biochem (1987) 56:89–124. 10.1146/annurev.bi.56.070187.000513 2441660

[28] Lieberman S Prasad V Heterodox notions on pathways of steroidogenesis. Endocr Rev (1990) 11(4):469–93. 10.1210/edrv-11-4-469 2292239

[29] Williamson M Sutcliffe M. Protein-protein interactions. Biochem Soc Trans (2010) 38(4):875–8. 10.1042/BST0380875 20658969

[30] An S Kumar R Sheets ED Benkovic SJ. Reversible compartmentalization of de novo purine biosynthetic complexes in living cells. Science (2008) 320(5872):103–6. 10.1126/science.1152241 18388293

[31] Deng Y Gam J French JB Zhao H An S Benkovic SJ. Mapping protein-protein proximity in the purinosome. J Biol Chem (2012) 287(43):36201–7. 10.1074/jbc.M112.407056 22955281 PMC3476287

[32] Lee K Kerner J Hoppel C. Mitochondrial carnitine palmitoyltransferase 1a (CPT1a) is part of an outer membrane fatty acid transfer complex. J Biol Chem (2011) 286(29):25655–62. 10.1074/jbc.M111.228692 21622568 PMC3138250

[33] Rone MB Midzak AS Martinez-Arguelles DB Fan J Ye X Blonder J Steroidogenesis in MA-10 mouse Leydig cells is altered via fatty acid import into the mitochondria. Biol Reprod (2014) 91(4):96. 10.1095/biolreprod.114.121434 25210128 PMC4435033

[34] Nebert D Wikvall K Miller W. Human cytochromes P450 in health and disease. Philos Trans R Soc Lond B Biol Sci (2013) 368(1612):20120431. 10.1098/rstb.2012.0431 23297354 PMC3538421

[35] Miller W. Genetic disorders of Vitamin D biosynthesis and degradation. J Steroid Biochem Mol Biol (2016). 10.1016/j.jsbmb.2016.04.001 27060335

[36] Heverin M Ali Z Olin M Tillander V Joibari MM Makoveichuk E On the regulatory importance of 27-hydroxycholesterol in mouse liver. J Steroid Biochem Mol Biol (2016). 10.1016/j.jsbmb.2016.02.001 26851362

[37] Nelson D Goldstone J Stegeman J. The cytochrome P450 genesis locus: the origin and evolution of animal cytochrome P450s. Philos Trans R Soc Lond B Biol Sci (2013) 368(1612):20120474. 10.1098/rstb.2012.0474 23297357 PMC3538424

[38] Markov GV Tavares R Dauphin-Villemant C Demeneix BA Baker ME Laudet V. Independent elaboration of steroid hormone signaling pathways in metazoans. Proc Natl Acad Sci U S A (2009) 106(29):11913–8. 10.1073/pnas.0812138106 19571007 PMC2715501

[39] Baker M. Origin and diversification of steroids: co-evolution of enzymes and nuclear receptors. Mol Cell Endocrinol (2011) 334(1–2):14–20. 10.1016/j.mce.2010.07.013 20654689

[40] Baker M Nelson D Studer R. Origin of the response to adrenal and sex steroids: roles of promiscuity and co-evolution of enzymes and steroid receptors. J Steroid Biochem Mol Biol (2015) 151:12–24. 10.1016/j.jsbmb.2014.10.020 25445914

[41] Johnson M Zaretskaya I Raytselis Y Merezhuk Y McGinnis S Madden TL. NCBI BLAST: a better web interface. Nucleic Acids Res (2008) 36(Web Server issue):W5–9. 10.1093/nar/gkn201 18440982 PMC2447716

[42] Edgar R. MUSCLE: multiple sequence alignment with high accuracy and high throughput. Nucleic Acids Res (2004) 32(5):1792–7. 10.1093/nar/gkh340 15034147 PMC390337

[43] Guindon S Gascuel O. A simple, fast, and accurate algorithm to estimate large phylogenies by maximum likelihood. Syst Biol (2003) 52(5):696–704. 10.1080/10635150390235520 14530136

[44] Payne A Hales D. Overview of steroidogenic enzymes in the pathway from cholesterol to active steroid hormones. Endocr Rev (2004) 25(6):947–70. 10.1210/er.2003-0030 15583024

[45] Shikita M Hall P. The stoichiometry of the conversion of cholesterol and hydroxycholesterols to pregnenolone (3beta-hydroxypregn-5-en-20-one) catalysed by adrenal cytochrome P-450. Proc Natl Acad Sci U S A (1974) 71(4):1441–5. 10.1073/pnas.71.4.1441 4151518 PMC388245

[46] Mast N Annalora AJ Lodowski DT Palczewski K Stout CD Pikuleva IA. Structural basis for three-step sequential catalysis by the cholesterol side chain cleavage enzyme CYP11A1. J Biol Chem (2011) 286(7):5607–13. 10.1074/jbc.M110.188433 21159775 PMC3037674

[47] Strushkevich N MacKenzie F Cherkesova T Grabovec I Usanov S Park HW. Structural basis for pregnenolone biosynthesis by the mitochondrial monooxygenase system. Proc Natl Acad Sci U S A (2011) 108(25):10139–43. 10.1073/pnas.1019441108 21636783 PMC3121847

[48] Davydov R Gilep AA Strushkevich NV Usanov SA Hoffman BM. Compound I is the reactive intermediate in the first monooxygenation step during conversion of cholesterol to pregnenolone by cytochrome P450scc: EPR/ENDOR/cryoreduction/annealing studies. J Am Chem Soc (2012) 134(41):17149–56. 10.1021/ja3067226 23039857 PMC3491644

[49] Davydov R Strushkevich N Smil D Yantsevich A Gilep A Usanov S Evidence that compound I is the active species in both the hydroxylase and lyase steps by which P450scc converts cholesterol to pregnenolone: EPR/ENDOR/cryoreduction/annealing studies. Biochemistry (2015) 54(48):7089–97. 10.1021/acs.biochem.5b00903 26603348 PMC4732517

[50] Lavoie H King S. Transcriptional regulation of steroidogenic genes: StARD1, CYP11A1 and HSD3B. Exp Biol Med (Maywood) (2009) 234(8):880–907. 10.3181/0903-MR-97 19491374

[51] John ME John MC Ashley P MacDonald RJ Simpson ER Waterman MR. Identification and characterization of cDNA clones specific for cholesterol side-chain cleavage cytochrome P-450. Proc Natl Acad Sci U S A (1984) 81(18):5628–32. 10.1073/pnas.81.18.5628 6592578 PMC391763

[52] Lebrethon MC Jaillard C Defayes G Begeot M Saez JM. Human cultured adrenal fasciculata-reticularis cells are targets for angiotensin-II: effects on cytochrome P450 cholesterol side-chain cleavage, cytochrome P450 17 alpha-hydroxylase, and 3 beta-hydroxysteroid-dehydrogenase messenger ribonucleic acid and proteins and on steroidogenic responsiveness to corticotropin and angiotensin-II. J Clin Endocrinol Metab (1994) 78(5):1212–9. 8175981 10.1210/jcem.78.5.8175981

[53] Guo IC Huang CY Wang CK Chung BC. Activating protein-1 cooperates with steroidogenic factor-1 to regulate 3’,5’-cyclic adenosine 5’-monophosphate-dependent human CYP11A1 transcription in vitro and in vivo. Endocrinology (2007) 148(4):1804–12. 10.1210/en.2006-0938 17218410

[54] Suzuki J Otsuka F Inagaki K Takeda M Ogura T Makino H. Novel action of activin and bone morphogenetic protein in regulating aldosterone production by human adrenocortical cells. Endocrinology (2004) 145(2):639–49. 10.1210/en.2003-0968 14592955

[55] Moore C Brentano S Miller W. Human P450scc gene transcription is induced by cyclic AMP and repressed by 12-O-tetradecanoylphorbol-13- acetate and A23187 through independent cis elements. Mol Cell Biol (1990) 10(11):6013–23. 10.1128/MCB.10.11.6013 1700277 PMC361399

[56] Lala D Rice D Parker K. Steroidogenic factor I, a key regulator of steroidogenic enzyme expression, is the mouse homolog of fushi tarazu-factor I. Mol Endocrinol (1992) 6(8):1249–58. 10.1210/me.6.8.1249 1406703

[57] Liu Z Simpson E. Molecular mechanism for cooperation between Sp1 and steroidogenic factor-1 (SF-1) to regulate bovine CYP11A gene expression. Mol Cell Endocrinol (1999) 153(1–2):183–96. 10.1016/S0303-7207(99)00036-2 10459866

[58] Monté D DeWitte F Hum D. Regulation of the human P450scc gene by steroidogenic factor 1 is mediated by CBP/p300. J Biol Chem (1998) 273(8):4585–91. 10.1074/jbc.273.8.4585 9468515

[59] Mornet E Dupont J Vitek A White PC. Characterization of two genes encoding human steroid 11 beta-hydroxylase (P-450(11) beta). J Biol Chem (1989) 264(35):20961–7. 2592361

[60] Midzak A Rone M Aghazadeh Y Culty M Papadopoulos V. Mitochondrial protein import and the genesis of steroidogenic mitochondria. Mol Cell Endocrinol (2011) 336(1–2):70–9. 10.1016/j.mce.2010.12.007 21147195 PMC3057322

[61] Nishimoto K Nakagawa K Li D Kosaka T Oya M Mikami S Adrenocortical zonation in humans under normal and pathological conditions. J Clin Endocrinol Metab (2010) 95(5):2296–305. 10.1210/jc.2009-2010 20200334

[62] Mulatero P Curnow KM Aupetit-Faisant B Foekling M Gomez-Sanchez C Veglio F Recombinant CYP11B genes encode enzymes that can catalyze conversion of 11-deoxycortisol to cortisol, 18-hydroxycortisol, and 18-oxocortisol. J Clin Endocrinol Metab (1998) 83(11):3996–4001. 10.1210/jcem.83.11.5237 9814482

[63] Ogishima T Shibata H Shimada H Mitani F Suzuki H Saruta T Aldosterone synthase cytochrome P-450 expressed in the adrenals of patients with primary aldosteronism. J Biol Chem (1991) 266(17):10731–4. 2040591

[64] Curnow KM Tusie-Luna MT Pascoe L Natarajan R Gu JL Nadler JL The product of the CYP11B2 gene is required for aldosterone biosynthesis in the human adrenal cortex. Mol Endocrinol (1991) 5(10):1513–22. 10.1210/mend-5-10-1513 1775135

[65] Kawamoto T Mitsuuchi Y Toda K Yokoyama Y Miyahara K Miura S Role of steroid 11 beta-hydroxylase and steroid 18-hydroxylase in the biosynthesis of glucocorticoids and mineralocorticoids in humans. Proc Natl Acad Sci U S A (1992) 89(4):1458–62. 10.1073/pnas.89.4.1458 1741400 PMC48470

[66] Yanagibashi K Haniu M Shively JE Shen WH Hall P. The synthesis of aldosterone by the adrenal cortex. Two zones (fasciculata and glomerulosa) possess one enzyme for 11 beta-, 18-hydroxylation, and aldehyde synthesis. J Biol Chem (1986) 261(8):3556–62. 3485096

[67] Morohashi K Yoshioka H Gotoh O Okada Y Yamamoto K Miyata T Molecular cloning and nucleotide sequence of DNA of mitochondrial cytochrome P-450(11 beta) of bovine adrenal cortex. J Biochem (1987) 102(3):559–68. 3429448 10.1093/oxfordjournals.jbchem.a122089

[68] Mellon S Bair S Monis H. P450c11B3 mRNA, transcribed from a third P450c11 gene, is expressed in a tissue-specific, developmentally, and hormonally regulated fashion in the rodent adrenal and encodes a protein with both 11-hydroxylase and 18-hydroxylase activities. J Biol Chem (1995) 270(4):1643–9. 10.1074/jbc.270.4.1643 7829497

[69] Chua SC Szabo P Vitek A Grzeschik KH John M White PC. Cloning of cDNA encoding steroid 11 beta-hydroxylase (P450c11). Proc Natl Acad Sci U S A (1987) 84(20):7193–7. 10.1073/pnas.84.20.7193 3499608 PMC299256

[70] Lifton RP Dluhy RG Powers M Rich GM Cook S Ulick S A chimaeric 11 beta-hydroxylase/aldosterone synthase gene causes glucocorticoid-remediable aldosteronism and human hypertension. Nature (1992) 355(6357):262–5. 10.1038/355262a0 1731223

[71] Kramer RE Rainey WE Funkenstein B Dee A Simpson ER Waterman MR. Induction of synthesis of mitochondrial steroidogenic enzymes of bovine adrenocortical cells by analogs of cyclic AMP. J Biol Chem (1984) 259(2):707–13. 6319383

[72] Denner K Rainey WE Pezzi V Bird IM Bernhardt R Mathis JM. Differential regulation of 11 beta-hydroxylase and aldosterone synthase in human adrenocortical H295R cells. Mol Cell Endocrinol (1996) 121(1):87–91. 10.1016/0303-7207(96)03853-1 8865169

[73] Wang XL Bassett M Zhang Y Yin S Clyne C White PC Transcriptional regulation of human 11beta-hydroxylase (hCYP11B1). Endocrinology (2000) 141(10):3587–94. 10.1210/en.141.10.3587 11014212

[74] Wang Z Bassett M Rainey W. Liver receptor homologue-1 is expressed in the adrenal and can regulate transcription of 11 beta-hydroxylase. J Mol Endocrinol (2001) 27(2):255–8. 10.1677/jme.0.0270255 11564608

[75] Clyne CD Zhang Y Slutsker L Mathis JM White PC Rainey WE. Angiotensin II and potassium regulate human CYP11B2 transcription through common cis-elements. Mol Endocrinol (1997) 11(5):638–49. 10.1210/mend.11.5.9920 9139807

[76] Nanba K Chen A Nishimoto K Rainey WE. Role of Ca(2+)/calmodulin-dependent protein kinase kinase in adrenal aldosterone production. Endocrinology (2015) 156(5):1750–6. 10.1210/en.2014-1782 25679868 PMC4398758

[77] Bassett MH Zhang Y Clyne C White PC Rainey WE. Differential regulation of aldosterone synthase and 11beta-hydroxylase transcription by steroidogenic factor-1. J Mol Endocrinol (2002) 28(2):125–35. 10.1677/jme.0.0280125 11932209

[78] Ye P Nakamura Y Lalli E Rainey WE. Differential effects of high and low steroidogenic factor-1 expression on CYP11B2 expression and aldosterone production in adrenocortical cells. Endocrinology (2009) 150(3):1303–9. 10.1210/en.2008-0667 18974272 PMC2654740

[79] Kurihara I Shibata H Kobayashi S Suda N Ikeda Y Yokota K Ubc9 and protein inhibitor of activated STAT 1 activate chicken ovalbumin upstream promoter-transcription factor I-mediated human CYP11B2 gene transcription. J Biol Chem (2005) 280(8):6721–30. 10.1074/jbc.M411820200 15611122

[80] Ewen KM Hannemann F Iametti S Morleo A Bernhardt R. Functional characterization of Fdx1: evidence for an evolutionary relationship between P450-type and ISC-type ferredoxins. J Mol Biol (2011) 413(5):940–51. 10.1016/j.jmb.2011.09.010 21945528

[81] Ziegler GA Vonrhein C Hanukoglu I Schulz GE. The structure of adrenodoxin reductase of mitochondrial P450 systems: electron transfer for steroid biosynthesis. J Mol Biol (1999) 289(4):981–90. 10.1006/jmbi.1999.2807 10369776

[82] Heinz A Hannemann F Müller JJ Heinemann U Bernhardt R. The interaction domain of the redox protein adrenodoxin is mandatory for binding of the electron acceptor CYP11A1, but is not required for binding of the electron donor adrenodoxin reductase. Biochem Biophys Res Commun (2005) 338(1):491–8. 10.1016/j.bbrc.2005.08.077 16137649

[83] Brentano ST Black SM Lin D Miller WL. cAMP post-transcriptionally diminishes the abundance of adrenodoxin reductase mRNA. Proc Natl Acad Sci U S A (1992) 89(9):4099–103. 10.1073/pnas.89.9.4099 1315050 PMC525640

[84] Harikrishna JA Black SM Szklarz GD Miller WL. Construction and function of fusion enzymes of the human cytochrome P450scc system. DNA Cell Biol (1993) 12(5):371–9. 10.1089/dna.1993.12.371 8517924

[85] Imamichi Y Mizutani T Ju Y Matsumura T Kawabe S Kanno M Transcriptional regulation of human ferredoxin reductase through an intronic enhancer in steroidogenic cells. Biochim Biophys Acta (2014) 1839(1):33–42. 10.1016/j.bbagrm.2013.11.005 24321386

[86] Müller A Müller JJ Muller YA Uhlmann H Bernhardt R Heinemann U. New aspects of electron transfer revealed by the crystal structure of a truncated bovine adrenodoxin, Adx(4-108). Structure (1998) 6(3):269–80. 10.1016/S0969-2126(98)00031-8 9551550

[87] Sheftel AD Stehling O Pierik AJ Elsässer HP Mühlenhoff U Webert H Humans possess two mitochondrial ferredoxins, Fdx1 and Fdx2, with distinct roles in steroidogenesis, heme, and Fe/S cluster biosynthesis. Proc Natl Acad Sci U S A (2010) 107(26):11775–80. 10.1073/pnas.1004250107 20547883 PMC2900682

[88] Davydov DR Davydova NY Sineva EV Halpert JR. Interactions among cytochromes P450 in microsomal membranes: oligomerization of cytochromes P450 3A4, 3A5, and 2E1 and its functional consequences. J Biol Chem (2015) 290(6):3850–64. 10.1074/jbc.M114.615443 25533469 PMC4319048

[89] Szczesna-Skorupa E Mallah B Kemper B. Fluorescence resonance energy transfer analysis of cytochromes P450 2C2 and 2E1 molecular interactions in living cells. J Biol Chem (2003) 278(33):31269–76. 10.1074/jbc.M301489200 12766165

[90] Rone MB Midzak AS Issop L Rammouz G Jagannathan S Fan J Identification of a dynamic mitochondrial protein complex driving cholesterol import, trafficking, and metabolism to steroid hormones. Mol Endocrinol (2012) 26(11):1868–82. 10.1210/me.2012-1159 22973050 PMC5416962

[91] Liu J Rone M Papadopoulos V. Protein-protein interactions mediate mitochondrial cholesterol transport and steroid biosynthesis. J Biol Chem (2006) 281(50):38879–93. 10.1074/jbc.M608820200 17050526

[92] Aghazadeh Y Rone MB Blonder J Ye X Veenstra TD Hales DB Hormone-induced 14-3-3γ adaptor protein regulates steroidogenic acute regulatory protein activity and steroid biosynthesis in MA-10 Leydig cells. J Biol Chem (2012) 287(19):15380–94. 10.1074/jbc.M112.339580 22427666 PMC3346089

[93] Aghazadeh Y Martinez-Arguelles DB Fan J Culty M Papadopoulos V. Induction of androgen formation in the male by a TAT-VDAC1 fusion peptide blocking 14-3-3ε protein adaptor and mitochondrial VDAC1 interactions. Mol Ther (2014) 22(10):1779–91. 10.1038/mt.2014.116 24947306 PMC4428399

[94] Poderoso C Converso DP Maloberti P Duarte A Neuman I Galli S A mitochondrial kinase complex is essential to mediate an ERK1/2-dependent phosphorylation of a key regulatory protein in steroid biosynthesis. PLoS One (2008) 3(1):e1443. 10.1371/journal.pone.0001443 18197253 PMC2175533

[95] Colombini M Mannella C. VDAC, the early days. Biochim Biophys Acta (2012) 1818(6):1438–43. 10.1016/j.bbamem.2011.11.014 22120576 PMC3296906

[96] Mertins B Psakis G Essen L. Voltage-dependent anion channels: the wizard of the mitochondrial outer membrane. Biol Chem (2014) 395(12):1435–42. 10.1515/hsz-2014-0203 25153596

[97] Blachly-Dyson E Baldini A Litt M McCabe ER Forte M. Human genes encoding the voltage-dependent anion channel (VDAC) of the outer mitochondrial membrane: mapping and identification of two new isoforms. Genomics (1994) 20(1):62–7. 10.1006/geno.1994.1127 7517385

[98] Messina A Reina S Guarino F De Pinto V VDAC isoforms in mammals. Biochim Biophys Acta (2012) 1818(6):1466–76. 10.1016/j.bbamem.2011.10.005 22020053

[99] Raghavan A Sheiko T Graham BH Craigen WJ. Voltage-dependant anion channels: novel insights into isoform function through genetic models. Biochim Biophys Acta (2012) 1818(6):1477–85. 10.1016/j.bbamem.2011.10.019 22051019 PMC4273737

[100] Baines CP Kaiser RA Sheiko T Craigen WJ Molkentin JD. Voltage-dependent anion channels are dispensable for mitochondrial-dependent cell death. Nat Cell Biol (2007) 9(5):550–5. 10.1038/ncb1575 17417626 PMC2680246

[101] Mannella C Forte M Colombini M. Toward the molecular structure of the mitochondrial channel, VDAC. J Bioenerg Biomembr (1992) 24(1):7–19. 10.1007/BF00769525 1380507

[102] Campbell A Chan S. The voltage dependent anion channel affects mitochondrial cholesterol distribution and function. Arch Biochem Biophys (2007) 466(2):203–10. 10.1016/j.abb.2007.06.012 17662230

[103] Hiller S Garces RG Malia TJ Orekhov VY Colombini M Wagner G. Solution structure of the integral human membrane protein VDAC-1 in detergent micelles. Science (2008) 321(5893):1206–10. 10.1126/science.1161302 18755977 PMC2579273

[104] Bayrhuber M Meins T Habeck M Becker S Giller K Villinger S Structure of the human voltage-dependent anion channel. Proc Natl Acad Sci U S A (2008) 105(40):15370–5. 10.1073/pnas.0808115105 18832158 PMC2557026

[105] Anholt RR De Souza EB Oster-Granite ML Snyder SH. Peripheral-type benzodiazepine receptors: autoradiographic localization in whole-body sections of neonatal rats. J Pharmacol Exp Ther (1985) 233(2):517–26. 2987488

[106] Braestrup C Squires R. Specific benzodiazepine receptors in rat brain characterized by high-affinity (3H)diazepam binding. Proc Natl Acad Sci U S A (1977) 74(9):3805–9. 10.1073/pnas.74.9.3805 20632 PMC431738

[107] Gavish M Bachman I Shoukrun R Katz Y Veenman L Weisinger G Enigma of the peripheral benzodiazepine receptor. Pharmacol Rev (1999) 51(4):629–50. 10581326

[108] Krueger K Papadopoulos V. Peripheral-type benzodiazepine receptors mediate translocation of cholesterol from outer to inner mitochondrial membranes in adrenocortical cells. J Biol Chem (1990) 265(25):15015–22. 2168398

[109] Papadopoulos V Mukhin AG Costa E Krueger KE. The peripheral-type benzodiazepine receptor is functionally linked to Leydig cell steroidogenesis. J Biol Chem (1990) 265(7):3772–9. 2154488

[110] Lacapere J Papadopoulos V. Peripheral-type benzodiazepine receptor: structure and function of a cholesterol-binding protein in steroid and bile acid biosynthesis. Steroids (2003) 68(7–8):569–85. 10.1016/S0039-128X(03)00101-6 12957662

[111] Rupprecht R Rammes G Eser D Baghai TC Schüle C Nothdurfter C Translocator protein (18 kD) as target for anxiolytics without benzodiazepine-like side effects. Science (2009) 325(5939):490–3. 10.1126/science.1175055 19541954

[112] Papadopoulos V Baraldi M Guilarte TR Knudsen TB Lacapère JJ Lindemann P Translocator protein (18kDa): new nomenclature for the peripheral-type benzodiazepine receptor based on its structure and molecular function. Trends Pharmacol Sci (2006) 27(8):402–9. 10.1016/j.tips.2006.06.005 16822554

[113] Joseph-Liauzun E Delmas P Shire D Ferrara P. Topological analysis of the peripheral benzodiazepine receptor in yeast mitochondrial membranes supports a five-transmembrane structure. J Biol Chem (1998) 273(4):2146–52. 10.1074/jbc.273.4.2146 9442055

[114] Li F Liu J Zheng Y Garavito RM Ferguson-Miller S Protein structure. Crystal structures of translocator protein (TSPO) and mutant mimic of a human polymorphism. Science (2015) 347(6221):555–8. 10.1126/science.1260590 25635101 PMC5125025

[115] Guo Y Kalathur RC Liu Q Kloss B Bruni R Ginter C Protein structure. Structure and activity of tryptophan-rich TSPO proteins. Science (2015) 347(6221):551–5. 10.1126/science.aaa1534 25635100 PMC4341906

[116] Lacapère JJ Delavoie F Li H Péranzi G Maccario J Papadopoulos V Structural and functional study of reconstituted peripheral benzodiazepine receptor. Biochem Biophys Res Commun (2001) 284(2):536–41. 10.1006/bbrc.2001.4975 11394915

[117] Li H Papadopoulos V. Peripheral-type benzodiazepine receptor function in cholesterol transport. Identification of a putative cholesterol recognition/interaction amino acid sequence and consensus pattern. Endocrinology (1998) 139(12):4991–7. 10.1210/en.139.12.4991 9832438

[118] Fan J Papadopoulos V. Evolutionary origin of the mitochondrial cholesterol transport machinery reveals a universal mechanism of steroid hormone biosynthesis in animals. PLoS One (2013) 8(10):e76701. 10.1371/journal.pone.0076701 24124589 PMC3790746

[119] Li H Yao Z Degenhardt B Teper G Papadopoulos V. Cholesterol binding at the cholesterol recognition/interaction amino acid consensus (CRAC) of the peripheral-type benzodiazepine receptor and inhibition of steroidogenesis by an HIV TAT-CRAC peptide. Proc Natl Acad Sci U S A (2001) 98(3):1267–72. 10.1073/pnas.98.3.1267 11158628 PMC14743

[120] Midzak A Akula N Lecanu L Papadopoulos V. Novel androstenetriol interacts with the mitochondrial translocator protein and controls steroidogenesis. J Biol Chem (2011) 286(11):9875–87. 10.1074/jbc.M110.203216 21209087 PMC3058962

[121] Midzak AS Akula N Rone MB Papadopoulos V. Computational modeling and biological validation of novel non-steroidal ligands for the cholesterol recognition/interaction amino acid consensus (CRAC) motif of the mitochondrial translocator protein (TSPO). Pharmacol Res (2015) 99:393–403. 10.1016/j.phrs.2015.03.023 25936508

[122] Costa B Pini S Gabelloni P Da Pozzo E Abelli M Lari L The spontaneous Ala147Thr amino acid substitution within the translocator protein influences pregnenolone production in lymphomonocytes of healthy individuals. Endocrinology (2009) 150(12):5438–45. 10.1210/en.2009-0752 19846611

[123] Boujrad N Vidic B Papadopoulos V. Acute action of choriogonadotropin on Leydig tumor cells: changes in the topography of the mitochondrial peripheral-type benzodiazepine receptor. Endocrinology (1996) 137(12):5727–30. 10.1210/en.137.12.5727 8940407

[124] Delavoie F Li H Hardwick M Robert JC Giatzakis C Péranzi G In vivo and in vitro peripheral-type benzodiazepine receptor polymerization: functional significance in drug ligand and cholesterol binding. Biochemistry (2003) 42(15):4506–19. 10.1021/bi0267487 12693947

[125] Culty M Li H Boujrad N Amri H Vidic B Bernassau JM In vitro studies on the role of the peripheral-type benzodiazepine receptor in steroidogenesis. J Steroid Biochem Mol Biol (1999) 69(1–6):123–30. 10.1016/S0960-0760(99)00056-4 10418986

[126] McEnery MW Snowman AM Trifiletti RR Snyder SH. Isolation of the mitochondrial benzodiazepine receptor: association with the voltage-dependent anion channel and the adenine nucleotide carrier. Proc Natl Acad Sci U S A (1992) 89(8):3170–4. 10.1073/pnas.89.8.3170 1373486 PMC48827

[127] Garnier M Dimchev AB Boujrad N Price JM Musto NA Papadopoulos V. In vitro reconstitution of a functional peripheral-type benzodiazepine receptor from mouse Leydig tumor cells. Mol Pharmacol (1994) 45(2):201–11. 8114671

[128] Gatliff J East D Crosby J Abeti R Harvey R Craigen W TSPO interacts with VDAC1 and triggers a ROS-mediated inhibition of mitochondrial quality control. Autophagy (2014) 10(12):2279–96. 10.4161/15548627.2014.991665 25470454 PMC4502750

[129] Tu LN Morohaku K Manna PR Pelton SH Butler WR Stocco DM Peripheral benzodiazepine receptor/translocator protein global knock-out mice are viable with no effects on steroid hormone biosynthesis. J Biol Chem (2014) 289(40):27444–54. 10.1074/jbc.M114.578286 24936060 PMC4183784

[130] Banati RB Middleton RJ Chan R Hatty CR Kam WW Quin C Positron emission tomography and functional characterization of a complete PBR/TSPO knockout. Nat Commun (2014) 5:5452. 10.1038/ncomms6452 25406832 PMC4263137

[131] Fan J Campioli E Midzak A Culty M Papadopoulos V. Conditional steroidogenic cell-targeted deletion of TSPO unveils a crucial role in viability and hormone-dependent steroid formation. Proc Natl Acad Sci U S A (2015) 112(23):7261–6. 10.1073/pnas.1502670112 26039990 PMC4466704

[132] Crivello J Jefcoate C Intracellular movement of cholesterol in rat adrenal cells. Kinetics and effects of inhibitors. J Biol Chem (1980) 255(17):8144–51. 6251046

[133] Pon L Hartigan J Orme-Johnson N. Acute ACTH regulation of adrenal corticosteroid biosynthesis. Rapid accumulation of a phosphoprotein. J Biol Chem (1986) 261(28):13309–16. 3020029

[134] Pon L Orme-Johnson N. Acute stimulation of steroidogenesis in corpus luteum and adrenal cortex by peptide hormones. Rapid induction of a similar protein in both tissues. J Biol Chem (1986) 261(14):6594–9. 3009462

[135] Arakane F King SR Du Y Kallen CB Walsh LP Watari H Phosphorylation of steroidogenic acute regulatory protein (StAR) modulates its steroidogenic activity. J Biol Chem (1997) 272(51):32656–62. 10.1074/jbc.272.51.32656 9405483

[136] Duarte A Castillo AF Podestá EJ Poderoso C. Mitochondrial fusion and ERK activity regulate steroidogenic acute regulatory protein localization in mitochondria. PLoS One (2014) 9(6):e100387. 10.1371/journal.pone.0100387 24945345 PMC4063759

[137] Bose HS Sugawara T Strauss JF III Miller WL International Congenital Lipoid Adrenal Hyperplasia Consortium. The pathophysiology and genetics of congenital lipoid adrenal hyperplasia. N Engl J Med (1996) 335(25):1870–8. 10.1056/NEJM199612193352503 8948562

[138] Arakane F Sugawara T Nishino H Liu Z Holt JA Pain D Steroidogenic acute regulatory protein (StAR) retains activity in the absence of its mitochondrial import sequence: implications for the mechanism of StAR action. Proc Natl Acad Sci U S A (1996) 93(24):13731–6. 10.1073/pnas.93.24.13731 8943003 PMC19407

[139] Bose H Lingappa V Miller W. Rapid regulation of steroidogenesis by mitochondrial protein import. Nature (2002) 417(6884):87–91. 10.1038/417087a 11986670

[140] Sasaki G Ishii T Jeyasuria P Jo Y Bahat A Orly J Complex role of the mitochondrial targeting signal in the function of steroidogenic acute regulatory protein revealed by bacterial artificial chromosome transgenesis in vivo. Mol Endocrinol (2008) 22(4):951–64. 10.1210/me.2007-0493 18187601 PMC2276465

[141] Baquedano MS Guercio G Marino R Berensztein E Costanzo M Bailez M Unique dominant negative mutation in the N-terminal mitochondrial targeting sequence of StAR, causing a variant form of congenital lipoid adrenal hyperplasia. J Clin Endocrinol Metab (2013) 98(1):E153–61. 10.1210/jc.2012-2865 23175692

[142] Bose M Whittal RM Miller WL Bose HS. Steroidogenic activity of StAR requires contact with mitochondrial VDAC1 and phosphate carrier protein. J Biol Chem (2008) 283(14):8837–45. 10.1074/jbc.M709221200 18250166 PMC2276375

[143] Gilquin B Taillebourg E Cherradi N Hubstenberger A Gay O Merle N The AAA+ ATPase ATAD3A controls mitochondrial dynamics at the interface of the inner and outer membranes. Mol Cell Biol (2010) 30(8):1984–96. 10.1128/MCB.00007-10 20154147 PMC2849464

[144] Da Cruz S Xenarios I Langridge J Vilbois F Parone PA Martinou JC. Proteomic analysis of the mouse liver mitochondrial inner membrane. J Biol Chem (2003) 278(42):41566–71. 10.1074/jbc.M304940200 12865426

[145] Issop L Fan J Lee S Rone MB Basu K Mui J Mitochondria-associated membrane formation in hormone-stimulated Leydig cell steroidogenesis: role of ATAD3. Endocrinology (2015) 156(1):334–45. 10.1210/en.2014-1503 25375035

[146] Hu J Prinz W Rapoport T. Weaving the web of ER tubules. Cell (2011) 147(6):1226–31. 10.1016/j.cell.2011.11.022 22153070 PMC3478066

[147] Martin L Kennedy B Karten B. Mitochondrial cholesterol: mechanisms of import and effects on mitochondrial function. J Bioenerg Biomembr (2016) 48(2):137–51. 10.1007/s10863-014-9592-6 25425472

[148] Brown M Goldstein J Cholesterol feedback: from Schoenheimer’s bottle to Scap’s MELADL. J Lipid Res (2009) 50(Suppl):S15–27. 10.1194/jlr.R800054-JLR200 18974038 PMC2674699

[149] Issop L Rone M Papadopoulos V. Organelle plasticity and interactions in cholesterol transport and steroid biosynthesis. Mol Cell Endocrinol (2013) 371(1–2):34–46. 10.1016/j.mce.2012.12.003 23246788

[150] Vance J. Phospholipid synthesis in a membrane fraction associated with mitochondria. J Biol Chem (1990) 265(13):7248–56. 2332429

[151] Mannella CA Buttle K Rath BK Marko M. Electron microscopic tomography of rat-liver mitochondria and their interaction with the endoplasmic reticulum. Biofactors (1998) 8(3–4):225–8. 10.1002/biof.5520080309 9914823

[152] Rizzuto R Pinton P Carrington W Fay FS Fogarty KE Lifshitz LM Close contacts with the endoplasmic reticulum as determinants of mitochondrial Ca2+ responses. Science (1998) 280(5370):1763–6. 10.1126/science.280.5370.1763 9624056

[153] Csordás G Várnai P Golenár T Roy S Purkins G Schneider TG Imaging interorganelle contacts and local calcium dynamics at the ER-mitochondrial interface. Mol Cell (2010) 39(1):121–32. 10.1016/j.molcel.2010.06.029 20603080 PMC3178184

[154] Raturi A Simmen T. Where the endoplasmic reticulum and the mitochondrion tie the knot: the mitochondria-associated membrane (MAM). Biochim Biophys Acta (2013) 1833(1):213–24. 10.1016/j.bbamcr.2012.04.013 22575682

[155] Szabadkai G Bianchi K Várnai P De Stefani D Wieckowski MR Cavagna D Chaperone-mediated coupling of endoplasmic reticulum and mitochondrial Ca2+ channels. J Cell Biol (2006) 175(6):901–11. 10.1083/jcb.200608073 17178908 PMC2064700

[156] Prasad M Kaur J Pawlak KJ Bose M Whittal RM Bose HS. Mitochondria-associated endoplasmic reticulum membrane (MAM) regulates steroidogenic activity via steroidogenic acute regulatory protein (StAR)-voltage-dependent anion channel 2 (VDAC2) interaction. J Biol Chem (2015) 290(5):2604–16. 10.1074/jbc.M114.605808 25505173 PMC4317014

[157] Marriott KS Prasad M Thapliyal V Bose HS. σ-1 receptor at the mitochondrial-associated endoplasmic reticulum membrane is responsible for mitochondrial metabolic regulation. J Pharmacol Exp Ther (2012) 343(3):578–86. 10.1124/jpet.112.198168 22923735 PMC3500540

[158] Hayashi T Su T. Sigma-1 receptors (sigma(1) binding sites) form raft-like microdomains and target lipid droplets on the endoplasmic reticulum: roles in endoplasmic reticulum lipid compartmentalization and export. J Pharmacol Exp Ther (2003) 306(2):718–25. 10.1124/jpet.103.051284 12730355

[159] Fujimoto M Hayashi T Su T. The role of cholesterol in the association of endoplasmic reticulum membranes with mitochondria. Biochem Biophys Res Commun (2012) 417(1):635–9. 10.1016/j.bbrc.2011.12.022 22185692 PMC3259743

[160] Lewin TM Kim JH Granger DA Vance JE Coleman RA. Acyl-CoA synthetase isoforms 1, 4, and 5 are present in different subcellular membranes in rat liver and can be inhibited independently. J Biol Chem (2001) 276(27):24674–9. 10.1074/jbc.M102036200 11319232

[161] Maloberti P Castilla R Castillo F Cornejo Maciel F Mendez CF Paz C Silencing the expression of mitochondrial acyl-CoA thioesterase I and acyl-CoA synthetase 4 inhibits hormone-induced steroidogenesis. FEBS J (2005) 272(7):1804–14. 10.1111/j.1742-4658.2005.04616.x 15794766

[162] Duarte A Poderoso C Cooke M Soria G Cornejo Maciel F Gottifredi V Mitochondrial fusion is essential for steroid biosynthesis. PLoS One (2012) 7(9):e45829. 10.1371/journal.pone.0045829 23029265 PMC3448708

